# Introduction to the Frontiers Research Topic: Optimization of Exercise Countermeasures for Human Space Flight – Lessons From Terrestrial Physiology and Operational Considerations

**DOI:** 10.3389/fphys.2019.00173

**Published:** 2019-03-07

**Authors:** Jonathan P. R. Scott, Tobias Weber, David A. Green

**Affiliations:** ^1^KBRwyle GmbH, Cologne, Germany; ^2^Space Medicine Team, European Astronaut Centre, European Space Agency, Cologne, Germany; ^3^Centre of Human and Applied Physiological Sciences, King’s College London, London, United Kingdom

**Keywords:** microgravity, exercise countermeasures, human space exploration, cardiovascular, musculoskeletal

## Abstract

Exercise in space has evolved from rudimental testing into the multi-modal countermeasure (CM) program used on the International Space Station (ISS). However, with the constraints of future exploration missions, replicating this program will be a significant challenge. Recent ISS data suggest that crew now experience only relatively moderate levels of microgravity (μG)-induced adaptation, although significant variation remains, with some crew displaying marked changes despite significant time/effort investment. This suggests that the efficacy of exercise CMs is yet to be optimized for *all* individuals. With the current suite of exercise devices operational for almost a decade, and with exploration approaching, it is timely to re-visit the terrestrial literature to identify new knowledge relevant to the management of μG adaptation. As such, the aim of the Frontiers Research Topic *Optimization of Exercise Countermeasures for Human Space Flight – Lessons from Terrestrial Physiology and Operational Considerations*, is to synthesize current terrestrial exercise physiology knowledge and consider how this might be employed to optimize the use of exercise CM. The purpose of this Perspective, which serves as a preface to the Research Topic is threefold: to briefly review the use and apparent efficacy of exercise in space, to consider the impact of the transition from ISS to exploration mission vehicles and habitats, and to identify areas of terrestrial exercise physiology where current knowledge might contribute to the optimization of CM exercise for exploration. These areas include individual variation, high intensity interval training, strength development/maintenance, concurrent training, plyometric/impact exercise, and strategies to enhance exercise efficacy.

## Introduction

Exposure to microgravity (μG) and the space environment results in a profound multi-system adaptation, characterized by both short- (Ortega and Harm, 2008) and long-term changes, including reductions in maximum oxygen uptake (VO_2max_), muscle size and strength, and bone mineral density (BMD) (Demontis et al., 2017). As these changes appear to reflect those that occur with prolonged inactivity or the absence of gravitational loading, since the early days of human spaceflight, physical exercise has been identified as a potential method of managing the adaptation process (Berry et al., 1962; Moore et al., 2010). Today, exercise is the cornerstone of the International Space Station (ISS) μG countermeasure (CM) program for long duration missions (LDMs), with approximately 25% of each working day allocated to aerobic and resistance exercise including time to change clothing, set-up and stow hardware, and post-exercise hygiene (Loehr et al., 2015).

Space agencies are turning their attention to human missions beyond Low Earth Orbit. Such missions, and the vehicles and habitats used to execute them, will place even tighter constraints upon the use of exercise, including working volume (e.g., size and internal dimensions), environmental (e.g., removal of CO_2_, heat and moisture), logistical (e.g., supply of food and water, and device maintenance/repair) and operational (e.g., time for exercise, interference with other crewmembers’ work) challenges. Some of these constraints are self-evident (e.g., smaller vehicles/working volumes) (Gerstenmaier and Crusan, 2018; National Aeronautics and Space Administration [NASA], 2018c), whereas others will emerge only once key technological hardware limitations are understood and mission scenarios clearly defined. Irrespective, it is clear that a direct transfer of the ISS exercise CM program to exploration missions will be challenging.

Terrestrial exercise physiology knowledge is constantly evolving, driven by both the investigation of new ideas and the accumulation of evidence that either supports or questions existing principles. The current ISS CM exercise program and the suite of devices around which it is based has been operational for almost a decade, and, with the dawn of human space exploration approaching, it is timely to re-visit the terrestrial literature to identify where this knowledge might inform the future use of exercise to manage μG adaptation.

The aim of this Perspective, which serves as a preface to the Frontiers Research Topic *Optimization of Exercise Countermeasures for Human Space Flight – Lessons from Terrestrial Physiology and Operational Considerations*, is threefold: to briefly review the use and apparent efficacy of exercise in space, to consider the impact of the transition from ISS to exploration mission vehicles and habitats, and identify potential areas where terrestrial exercise physiology knowledge might contribute to the optimization of future spaceflight CM exercise. The Research Topic will focus primarily on the United States (US) space program due to the availability of information from NASA concerning its historical programs and current US Orbital Segment (USOS) crew, and because is currently leading the way in the development of exploration transport vehicles (National Aeronautics and Space Administration [NASA], 2018c) and habitats (Gerstenmaier and Crusan, 2018).

‘Optimization’ in the context of exercise CM may be defined in a number of ways depending on the specific goal(s) of the program. These goals might include maintenance of pre-flight physical status (i.e., prevent adaptation), preservation of sufficient capacity to safely execute mission tasks and/or function immediately on landing, a rapid return to pre-flight status in the post-flight period, or to minimize the risk of long-term health consequences. Alternatively, an optimal CM could simply be the approach that achieves the greatest physiological effect in the largest proportion of the target population. However, for the purposes of this Perspective and of future human space exploration, ‘optimization’ is defined by the goals of:

(1) maintaining sufficient physiological function in *all* crew to achieve mission-specific tasks, both nominal and off-nominal/emergency, including those immediately on landing, without approaching the limits of their physical capacity;
(2) using an exercise program than requires minimal additional utilization of mission and life support system resources.

## The Use of Exercise in Space: A Brief History

For a comprehensive history of the use of aerobic exercise in space, the reader is directed to the review of Moore et al. (2010) and for a concise summary of the exercise hardware available during each era, to Hackney et al. (2015). Initially use of physical activity in the US space program sought to explore the physiological effects of the μG environment, and only later was it considered as a potential CM for musculoskeletal and cardiovascular adaptation. The first medical observations on humans were made during Project Mercury. Crewmembers performed rudimentary exercise tests by pulling on a bungee cord (16lb [7.25 kg] at full extension) whilst evaluating cardiovascular reactivity. Despite the short mission durations, some post-flight postural hypotension was observed on return to Earth, resulting in NASA’s Aerospace Medical Operations Office concluding in 1962 that a “*prescribed inflight exercise program may be necessary to preclude symptoms in case of the need for an emergency egress soon after landing*” (Berry et al., 1962).

The Gemini Program (1961–1966) provided the first series of studies detailing the physiological response to spaceflight during missions of up to 14-days. Exercise testing consisted of 30-s exercise sessions using a bungee pull cord device (Berry and Catterson, 1967). Post-flight testing of Gemini VII crew, suggested, albeit indirectly, that as little as 14-days in space significantly reduced aerobic exercise capacity (Dietlein and Rapp, 1966).

**FIGURE 1:**
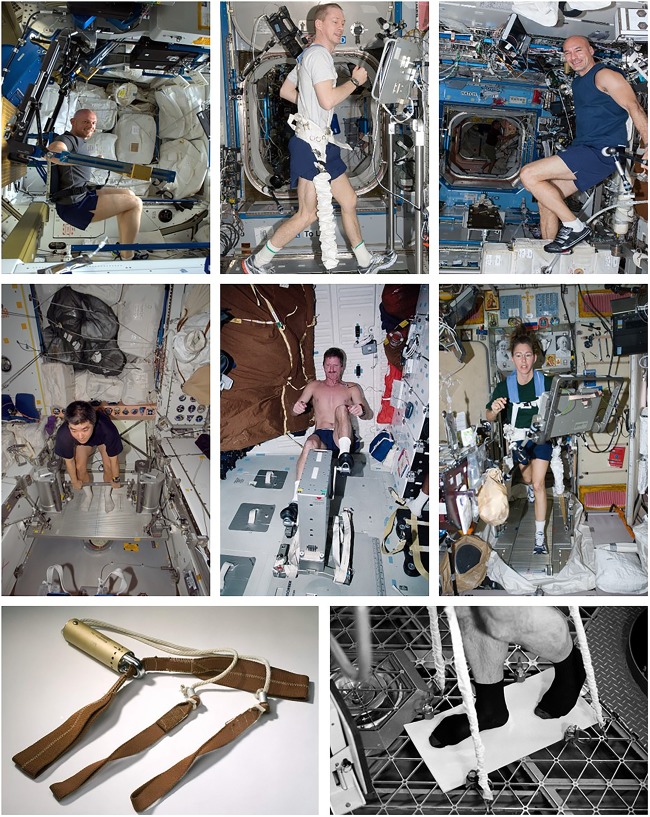
Hardware used for exercise countermeasures. *Top Row* (from left to right): ESA Astronaut Alexander Gerst exercising using the advanced resistive exercise device (ARED) on the International Space Station (ISS); Copyright: ESA/NASA: Id 312342); ESA Astronaut Frank de Winne using the T2 treadmill on ISS (Copyright: NASA: ISS021-E-007807); ESA Astronaut Luca Parmitano using the Cycle Ergometer with Vibration Isolation and Stabilization System (CEVIS) on ISS (Copyright: ESA/NASA: Id 300078). *Middle Row* (from left to right): NASA Astronaut Dan Tani, Expedition 16 Flight Engineer, using the Interim Resistive Exercise Device (iRED) on ISS (Copyright: NASA: ID iss016e027909); Astronaut Joseph Tanner, STS-97 Mission Specialist, using the cycle ergometer aboard the Space Shuttle Endeavour (Copyright: NASA: ID sts097-317-017); Astronaut Sandra Magnus, Expedition 18 Flight Engineer, equipped with a bungee harness, using the Treadmill with Vibration Isolation and Stabilization System (TVIS) in the Zvezda Service Module on ISS (Copyright: NASA: NASA ID iss018e030096). *Bottom Row* (from left to right): The ‘Apollo Exerciser’ used by Apollo 11 astronauts during their July 1969 mission (Photo by Eric F. Long, Smithsonian National Air and Space Museum [NASM 2009-4775] Used with Permission [Permission Number: 19-BK-063]); The Teflon-covered treadmill-like device used during Skylab 4 (Photo Credit: NASA).

The Apollo Program (1961–1972) was the first to use in-flight exercise as a countermeasure. Whilst no formal exercise program was planned, all crewmembers used (with varying frequency and intensity) the ‘Apollo Exerciser,’ a modified commercial-off-the-shelf variable resistance rope friction device (Figure 1) (Scheuring et al., 2007). The physiological benefits of such training are unclear (Orlee, 1973), although in-flight, crew reported that exercise helped with rest, relaxation, and stretching cramped and aching muscles (Scheuring et al., 2007).

Skylab (SL, 1973–1974) was utilized for three manned missions. SL-2 (28-days) crew were allocated 30 min/day for exercise and used the M171 cycle ergometer (286W maximum workload) (Michel et al., 1977), with protocols recommended, but not imposed (Sawin et al., 1975). On the recommendation of SL-2 crew, SL-3 (56-days) crew were allocated 60-min/day for exercise (Johnston, 1977). A modified commercial isokinetic device (“Mini-Gym” or MK-1) was also provided, as well as a pair of handles between which up to five extension springs could be attached (“MK-2”) (Thornton and Rummel, 1977). Exercise allowance during SL-4 (84-days) was further increased to 90-min/day (Johnston, 1977) and a rudimentary treadmill-like system provided, consisting of a Teflon-covered surface with rubber bungee restraints (Figure 1) (Thornton and Rummel, 1977). Due to high loads on the calf muscles, exercise was limited to 10 min/day of walking, jumping, or jogging.

Leg extensor strength was reduced by ∼25% in SL-2 (0.9%/day) and SL-3 (0.4%/day), and by <10% (0.1%/day) during SL-4, with crew standing and walking without apparent difficulty on the day after landing/recovery (R+1) (Thornton and Rummel, 1977). Heart rate at 75% maximum work rate was unchanged during SL-2, SL-3 (Michel et al., 1975) and SL-4 (Michel et al., 1977). Despite ergometer workload limitations, it was concluded that SL-4 crew maintained, or even increased their aerobic capacity (Sawin et al., 1975), whilst post-flight recovery of numerous cardiovascular parameters appeared more rapid from SL-2 to SL-4 (Michel et al., 1977).

Space Shuttle (135 flights, 1981–2011) missions ranged from 2 to 17 days. A cycle ergometer (Figure 1) was the primary exercise device, although two treadmills and a rower were also evaluated (Hackney et al., 2015). Flight Rules stated that exercise should be performed no less than every other day for the Commander, Pilot and Flight Engineer, and every third day for Mission and Payload Specialists, but intensity and duration were not prescribed (Lee et al., 2015). Peak oxygen uptake (VO_2peak_) was maintained during flights up to 14-days, but reduced 22% immediately post-flight (R+0), presumably due to reductions in blood volume, stroke volume and cardiac output (Levine et al., 1996).

The Extended Duration Orbiter Medical Project included comparisons of exercising and non-exercising Shuttle crew (Sawin et al., 1999). Individual exercise volume varied considerably, but whereas non-exercisers (and cycling only) showed a significant (12–13%) decrease in VO_2max_, treadmill (-3%) and rower (-6%) users showed little change, although the former tended to be tested sooner after landing. Compared with crew who exercised < 3 sessions/week, crew who exercised ≥ 3 sessions/week had a lower HR response and maintained pulse pressure during a post-flight standing (orthostatic) test (Lee et al., 1999). Additionally, crew who exercised at ≥ 70% maximum HR (HR_max_) demonstrated the smallest (-9 vs. -15 to -23%) reduction in VO_2_ during exercise at 85% HR_max_ (Hayes et al., 2013).

## The Current Use of Exercise on the ISS and Its Effectiveness in Managing Spaceflight Adaptation

For detailed overviews of the current USOS countermeasure program, the reader is directed to Hackney et al. (2015), Korth (2015), and Loehr et al. (2015). Briefly, the key characteristics are:

• Consists of both aerobic and resistance exercise;
• High-frequency program, consisting of two sessions/day (1x 30–45 min of aerobic and 1x 45-min of resistance), 6-days/week;
• Multi-modal, utilizing one resistance device [the Advanced Resistive Exercise Device, (ARED)] and two aerobic devices [a treadmill (T2), and the Cycle Ergometer with Vibration Isolation and Stabilization System, CEVIS] (Figure 1);
• Aerobic and resistance sessions are completed on the same day, sometimes with only a minimal break in-between;
• T2 allows running speeds up to 20.4 km/h (12.7 m/h) with vertical loads equivalent to 54.4–68.0 kg (120–150 lbs). In 2010, T2 replaced the Treadmill with Vibration Isolation and Stabilization System (TVIS) (Figure 1), which had a maximum speed of only 10 m/h (Korth, 2015) and produced foot forces that were substantially lower than during walking/running on Earth (Cavanagh et al., 2010; Genc et al., 2010);
• CEVIS provides workloads up to 350 W at 120 rpm (Danish Aerospace Company, 2018);
• Aerobic sessions consist of steady-state and interval-type protocols, with target intensities of 75–80 and 60–90% VO_2max_;
• ARED engages all major muscle groups, with loads up to 272 kg (600 lb). In 2009, ARED replaced the interim resistive exercise device (iRED) (Figure 1), which suffered from wide variations in load with position and rate of change of position, had its maximum load limited to 136 kg (Korth, 2015), and, like TVIS, resulted foot forces that were substantially less than on Earth (Cavanagh et al., 2010; Genc et al., 2010);
• Resistance protocols are multi-set, multi-repetition for the lower and upper body, with initial loads calculated from a 10-repetition maximum load (plus 75% of bodyweight to compensate for the absence of bodyweight) and adjusted thereafter based on actual performance.

A significant challenge in estimating exercise CM effectiveness is the absence of astronauts who have performed no CM exercise. On ISS, Flight Rules dictate that all LDM crewmembers perform exercise, which precludes abstinence and intervention studies with a ‘no exercise’ control group. As a result, the effectiveness of exercise CM can only be compared to previous missions (Sibonga et al., 2015), or to a time prior to a significant change in hardware, such as the replacement of iRED with ARED (English et al., 2015; Sibonga et al., 2015).

**FIGURE 2:**
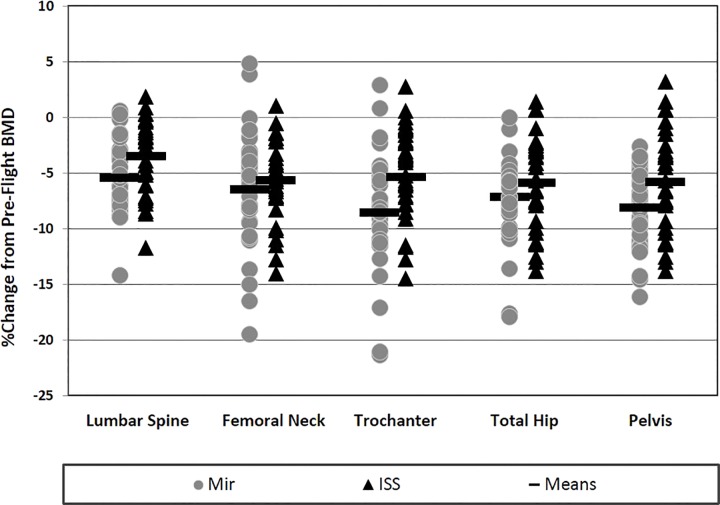
Inter-individual variation in changes in bone mineral density (BMD) with long-duration spaceflight. Depicted are relative (%) changes from pre- to post-flight in ISS (*n* = 33, triangles) and Mir (*n* = 35, circles) crewmembers. BMD was measured using Dual-energy X-ray absorptiometry (DXA). (Figure reprinted with permission from: *Evaluating Bone Loss in ISS Astronauts*, Sibonga et al., 2015).

In ISS crew, BMD loss is approximately 3% at the lumbar spine, 6% at the hip and pelvis, and significantly less than that measured in MIR crew (117–438 days missions) except at the femoral neck (Sibonga et al., 2015). However, individual variation is marked, with some crew still losing 10–15% (1.7–2.5%/month based on a 6-month mission), as shown in a recent paper (see Figure 2, Sibonga et al., 2015). Replacement of iRED with ARED has reduced bone loss at all sites (-2.6 to -4.1 vs. -3.7 to -6.6). Isokinetic muscle strength in the trunk and lower limbs is reduced by 8–17% post-flight (lower 95% confidence intervals from -12 to over -20%), whilst ARED has, as yet, not significantly attenuated strength decrements vs. iRED (English et al., 2015). Compared with pre-flight, mean VO_2peak_ during cycling declines 17% early in-flight and recovers only slightly thereafter (Moore et al., 2014), whilst being reduced by 15% (vs. pre-flight) on R+1. Again, there is significant individual variation, with some retaining their pre-flight VO_2peak_ at Flight Day (FD)180, whilst others lose up to 25%. In addition, whilst fitter crew who achieve higher exercise intensities appear to sustain their pre-flight VO_2peak_, they also appear more prone to the losses early in flight (Moore et al., 2014). On R+0 the proportion of ISS astronauts unable to complete an orthostatic tolerance test was 66% (4/6) compared with 20% (13/65) in Shuttle astronauts (Lee et al., 2015) and 83% (5/6) in Mir crew (Meck et al., 2001). Finally, in 10 tests of functional fitness including sit-and-reach, agility, calisthenics and strength, post-flight decrements at R+5-7 were seen only in sit-and-reach (-8%), agility (-11%), less press (-3%), and grip strength (-5%) (Laughlin et al., 2015).

## The Future Use of Exercise for Human Exploration Missions

As described above, the evolution of ISS exercise CM hardware has enabled the comprehensive (i.e., not limited in terms of duration, frequency, or intensity/overload) adoption of terrestrial exercise training concepts, including continuous and interval-type aerobic exercise and high-intensity, multi-set/rep resistance training. Training programes based on these concepts appear to result in, *on average*, relatively moderate levels of μG-induced musculoskeletal and cardiorespiratory system adaptation, although significant individual variation remains. It is evident, therefore, that the efficacy of ISS exercise CMs are yet to be optimized for *all* individuals. Moreover, with the constraints of future exploration missions, direct transfer of the ISS CM exercise program will be a significant challenge. NASA’s Orion vehicle has a habitable volume of less than 9 m^3^ (compared to 388 m^3^ on ISS) (National Aeronautics and Space Administration [NASA], 2018b,c), whilst the current concept of the Lunar Orbital Platform-Gateway, where crew may spend up to 30-days, envisions only two habitation modules, plus one utilization module (Gerstenmaier and Crusan, 2018). As a result, simply replicating the current efficacy of exercise CM during future exploration missions may be difficult. Furthermore, the size of these vehicles/habitats (limiting storage) and their remoteness from Earth (limiting re-supply) may, for the first time, require the burden of exercise on the supply of food (to maintain energy balance), and water (to maintain euhydration), as well as on the environmental management system’s regulation of atmospheric CO_2_, heat and moisture, to be considered (Matsuo et al., 2012; Scott et al., 2018). Taken together, it is clear that innovative approaches will be required.

A significant barrier in identifying new approaches is the limited opportunities to perform controlled intervention studies, both in space and in spaceflight analogs, of which long-duration head-down (typically -6°) bed rest (HDBR) is considered the ‘gold standard’ (Pavy-Le Traon et al., 2007; Hargens and Vico, 2016). Research in space is both costly and time-consuming, and NASA’s ‘SPRINT’ study (National Aeronautics and Space Administration [NASA], 2018a), which is evaluating a high intensity, low volume exercise training that has shown encouraging results in both HDBR (Ploutz-Snyder et al., 2018) and μG (Goetchius et al., 2019), is a rare example of a controlled, in-flight exercise training intervention study. Even here, however, the control group will not refrain from exercise, but continue to perform normal ISS CM exercise. Despite running since 2011, the recent publication of SPRINT results highlights the time-consuming nature of this type of research. Albeit less so than space studies, HDBR campaigns are also expensive and challenging, but offer greater experimental control and allow questions to be answered more quickly. However, at present, HDBR campaigns are organized at a rate of only 1–2 per year, are not exclusively focused on exercise CM, and, in the near future at least, may be subject to other priorities (e.g., artificial gravity) (Clément, 2017).

## A New Frontiers Research Topic

Almost a decade has past since the current suite of ISS exercise devices became operational, and with exploration missions approaching yet limited opportunities to evaluate new strategies, it is an opportune moment to re-visit the terrestrial exercise physiology literature to inform current and future exercise CM strategies. This literature includes a large number of controlled intervention studies in which exercise variables (i.e., mode, frequency, duration, workload, time-under-tension, recovery) have been controlled and systematically manipulated. This body of knowledge has expanded rapidly during the lifetime of the ISS and traditional exercise concepts have been re-visited and established beliefs challenged. As such, the literature may contain novel information to help identify strategies that may be both effective and compatible with exploration constraints. Topics identified as potentially conducive to the optimization of in-flight CM exercise include, but are not limited to:

• Individual variation: real variation vs. within-subject random variation (Atkinson and Batterham, 2015);
• High intensity interval training: efficacy and safety (Weston et al., 2014; Milanović et al., 2015);
• Strength development and maintenance: the contribution of different training variables to the effectiveness of resistance training (Ralston et al., 2017);
• Concurrent training: the scheduling of aerobic and resistance exercise for maximizing training gains (Wilson et al., 2012);
• ‘Combined training’: resistance exercise benefits across multiple physiological systems (Taylor et al., 2015; Paoli et al., 2017);
• Plyometric/impact exercise: effects on both the musculoskeletal *and* cardiovascular systems(Kramer et al., 2017);
• ‘Efficient’ training: is the efficacy of exercise training maintained when volume (e.g., duration, frequency) is reduced? (Metcalfe et al., 2012; Baker et al., 2013);
• The role of nutrition in promoting adaptations to exercise training (Hawley et al., 2011; Papageorgiou et al., 2018);
• Complementary strategies: CM that could enhance the effects of, or reduce reliance on, exercise (Carraro et al., 2015; Hackney et al., 2016).

These topics are to be evaluated initially in terms of the strength of the terrestrial evidence-base, then by potential operational advantages over current exercise CM approaches, and finally by the opportunities/challenges associated with integrating them into human spaceflight operations.

Therefore, the aim of the Frontiers Research Topic *Optimization of Exercise Countermeasures for Human Space Flight – Lessons from Terrestrial Physiology and Operational Considerations* is to synthesize current exercise physiology knowledge and consider how it might be employed to optimize the use of exercise to manage μG-induced adaptation.

## Author Contributions

JS, TW, and DG wrote and edited the article.

## Conflict of Interest Statement

All authors are employed by KBRWyle GbmH, Cologne, Germany.

## Abbreviations

ARED: Advanced Resistive Exercise Device
BMD: mean bone mineral density
CEVIS: cycle ergometer with vibration isolation and stabilization system
CM: countermeasure
CO_2_: carbon dioxide
ESA: European Space Agency
HDBR: head-down bed rest
HIIT: high-intensity interval training
HR: heart rate
HR_max_: maximum heart rate
iRED: interim resistive exercise device
ISS: international space station
LDM: long-duration mission
NASA: The National Aeronautics and Space Administration
TVIS: Treadmill Vibration Isolation System
US: United States
USOS: US Orbital Segment
VO_2max_: maximal oxygen uptake
VO_2peak_: peak oxygen uptake; μG, microgravity
